# Supplementary material for: Molecular and Cytogenetic Identification of Wheat-Thinopyrum intermedium Double Substitution Line-Derived Progenies for Stripe Rust Resistance
Source: Plants (Basel). 2022 Dec 21;12(1):28. doi: 10.3390/plants12010028 (PMC9823681; doi:10.3390/plants12010028)
Supplement: Supplementary file 1 [file plants-12-00028-s001.zip › plants-2041806-supplementary.pdf]

Table S1. The genome-specific oligo probes for distinguishing wheat and *Thinopyrum* chromatin by ND-FISH

Name	Sequences	References
Oligo-k288	CTTCATAGTCCGGGAGTCCGGCCAAAGGTCATAGTCCGGC CATCC	Wang et al. 2019
Oligo-D	TACGGGTGCCAAACGAGTGTCTGAAAGACTCCTCGAGAG GAAAATGCGAA	Tang et al. 2018
Oligo-pDb12H	TCAGAATTTTTAGGATAGCAGAAGTATTCGAAATACCCAG ATTGCTACAG	Yu et al. 2019, 2021
Oligo-B11	TCCGCTCACCTTGATGACAACATCAGGTGGAATTCCGTTC GAGGG	Xi et al. 2019
Oligo-5SrDNA	TCAGAACTCCGAAGTTAAGCGTGCTTGGGCGAGAGTAGTA C	Yu et al. 2019
Oligo-pTa71	GGGCAAAACCACGTACGTGGCACACGCCGCGTA	Tang et al. 2014
Oligo-(GAA)7	GAAGAAGAAGAAGAAGAAGAA	Lang et al. 2019
Oligo-CCS1	CCGTT TGATA GAGGCAAAGG TGTCC CGTCT TTTGATGAGA	Tang et al. 2014
Oligo-pSc119.2	CCGTTTTGTGGACTATTACTCACCGCTTTGGGGTCCCATAG CTAT	Tang et al. 2014
Oligo-pTa535	AAAAACTTGACGCACGTCACGTACAAATTGGACAAACTCT TTCGGAGTATCAGGGTTTC	Tang et al. 2014
Oligo-pSt122	GGCTCACATTAGGGAAGAATCGGTGAACAAAGAAAAGAC AAATTCACCGTATAGAG	Li et al. 2016
Oligo-744	GCCACCGTGC AGTAGACTTT TTTTGTACCC AAACCATCAG TAACAAAGTT CGTTCAC	This study

Table S2. The marker sequences for physical location of chromosome 4St-J^S

Marker	Forward	Reverse
CINAU1266	GAGAAGTCCAAGCAGAACAAAGA	GGCAGTACTTGTCCTGGATCT
CINAU1268	AACCCTGTGAATTATGGCCG	GTTCTCCTGTCTCTTCCTCCC
CINAU1269	AGAGGAAGAAGGACAAGGGC	CCATTCCAACACACACTGCA
CINAU1271	GTTGGTGATGAGGATGTTGCA	TCTGTTTGGTTCTGTGGGAGA

CINAU1274	GACATCATGCGCTGCTGG	GCGGCTGTTCCTTGATTGG
CINAU1275	AGCCTTTCCTGGTGGCATTA	GGGAAGATGAGACTTTCTTTGGG
CINAU1276	TGCTCTGACTGTCGGTGATT	AAGTTCAGCGGCAGTGAAAG
CINAU1279	AGTCAAACGCAGATTTCGTGA	CAGTCACCAATAACATGCCCA
CINAU1280	AATCACTCCTCCAGCCCCAA	CCTTGCATAGATACCAACCCG
CINAU1281	CCACCATCTGTCATTGATCGT	TCAAAGCGCGACCATACTTG
CINAU1282	GGCATCAGACGGAAGAATGG	ACGAATCGCATTGTAGATCTCT
CINAU1283	ACCTCACACACACTGAATCCA	CCGGAATGTAGCCCCTGG
CINAU1284	TCCAAGACCATGTACCTCGAG	GAACCTCTCCTGCCCAGC
CINAU1285	CCCCTCCGTGCTTACTACTA	TCCACAATGCCCTTAATGCC
CINAU1286	ACCACTCTCTCAGCTCAGC	TTCTGAAGAAAGCCCAGTTGT
CINAU1287	TGAACAATTACGACAAGTTCCTG	TGTCTGCAAGTTTACTGCCA
CINAU1288	AAAATTGCCACCTCTGCAGA	TGCTTAGGATCAGTTGTGCG
CINAU1289	GCCCTGCATTTGGTAATTGC	GTCAAAAGTGCCATAAATAGCCT
CINAU1291	GAGGAGAACACTGGATATATGCA	ACACTTTCCTATGCCACCTG
CINAU1292	AGAAGTGTACGTGTGAAGAATGA	TCCTTTCCAATGTCTTGCTCG
CINAU1295	GGTGGTAGTTCAAGCAAGACT	TCGCCTTGGAGATATGTCGT
CINAU1296	GGGTGGTGGTTGATGGCAA	CCTTGTACGCCTTGCAGATG
CINAU1297	TCTGCCCGGTCTCAACTATC	AGCACATTTGGTATCCTCGC
CINAU1299	TGGCACTGGAACTCAACTGT	CAACGAGTGCAAGCAGGAAT
CINAU1300	GTGGCTCCAAGTCCTCAGG	GCCTGCTGATATGGTGTGC
CINAU1301	GCGTGTTGATCTGCCTGAAA	TCCAGGTATCCTTGATGTTCCT
CINAU1302	CTGAAAGGCGCATTTCTGGT	TCCTTGGCGAAGATCACAGT
CINAU1303	AGAATGGCGCGAAGTAGAGA	GGCGGGGTTATCTTTGTCTTC
CINAU1304	CATCGGTCCCTACATTTGCG	CCGGAACATACTTCAGCCAG
CINAU1305	ATGTATTCCGCCACCAAACG	CTGGAGAACTGAGCGACGTA
CINAU1306	TGCATGCTTGGATGCTTTCT	CCTTGCACCTCGGACAGTA
CINAU1307	AGTTATTTCCTGGCCAGAAAACT	TTGGAATCAGCATCCAGGGA
CINAU1308	TCGGTAGATGGTGGATTGCA	CAACCTGGCCAGCTACATG

CINAU1309	CTCAGGCTGTCTATAGGTTGTTC	GTAGCCCACAAGTCGTTCCA
CINAU1310	TCTATGAACAAGGAAACCCGAA	GGTCTGTGAGGCTTCGGT
CINAU1311	CTTAACGCTCTGATGAAGGGA	AAGCACGTTCCTTCAGCAAG
CINAU1312	GCGGCGACATGAGATCTTTC	GCACATCTGGGTCCGTCAT
CINAU1314	ACGCATAAAGGCTCTTGGTG	TTGGCTAGACGAAATGCAGC
CINAU1317	CAGTTGGCCAAGAACCTAGC	GACGGGCGATGCTATCCA
CINAU1318	TGTTTTCACTGGCAAGCAAAA	ATGCTCTCCAGGGTTCTCAC
CINAU1319	TGACTTCTCTGGCAAAACGAG	AAGTTTCGCCATGCTTCTCC
CINAU1320	CTTGGACGAGCAATTACAGTG	GCTGTACAATGAACGCCACA
CINAU1321	CCACAACTTCCTCTTCTCCG	GACAAAGGTGCATTCATGTCA
CINAU1323	GGGTGCTTTACTTTGTTGAGGT	CATGCGCCTCCTTGAGTG
CINAU1324	TCAAACGAAATGCCCAGTCC	GCAAACGAGCAAACAGATCC
CINAU1325	TCTCGCTAACCTAAGGAGCA	GACGATCGGTGACAGGACAT
CINAU1326	CATTAAGATTTGGACACCTTCGT	CATCACCACCAGTTGCGAAA
CINAU1329	CAAACACGGATCAAAGAGGGG	ATGGGAAGGTCTGTACGCC
CINAU1330	GTTCACCGTCATGCTGAGG	CTCCACCTTGCTCCTCTCC
CINAU1331	AGCTCACAAAGAAGGAACAACA	TTCCGTCAAAACTCCAAGGC
CINAU1332	GGTATAGTCTGTGATTGCTGCA	CGTCTTTTCCCCATTCCAGC
CINAU1333	AGTGAGCCAGATATCCTTGACA	CCCAAGTTAGAGCATCTTCTGG
CINAU1334	AGTGGCTGTGTTAATTCATGCA	GTTGGAGAAACGAACGGGG
CINAU1336	CTGCCCCGAACCAGATACC	AGTAGTTCCTGCCATCCACC
CINAU1337	TGGCCGAAATCAAAGAAGCC	CGCGGCTACATAAGGGAGAT
CINAU1338	CACAGTATGAATTTGGTGCCAA	GCCTTCCATCAGTCATCAACC
CINAU1339	GATTGGACCGTGGCCTTG	AGATACTGACAGCTTCGACTG
CINAU1340	TCCTCTACAGTCAAGCGCTC	CCGAAAATTTCCGAGAGTAGTGA
CINAU1341	GCTTCACCACCTTTGCTCTC	CTGTCCAGGGAGCCATCAG
CINAU1342	CGAGAGAGCGCTGATTGTAG	GAGCTTCTCAATTTCCAGGACC
CINAU1343	TGGAGATAGAGCGGGAGAAAC	AGCGAGCATCTTCAAGAGGA
CINAU1344	CAGTGCCTCGACCTCCAA	CCTGTTCCAGCTCCTCTTCA

CINAU1345	ACCGACTCACAACTGCAATG	TGTTCCATTCTCCAGGACGA
CINAU1346	ACAGGAATTTGTTAGAGGAGCT	AAATTGCTCTGTCTGAAAACGT
CINAU1347	CTGGGTCATGCACGAGTTC	GTGAGCTCTTTGTTGAACACCT
CINAU1348	TCGAGCTGAAGAAATACGGGA	AGGAGGCATCATCCACGAC
CINAU1349	AGAAAGGCAAAATGGAGGACA	ACTCAACAAGGCTAGTCAAACC
CINAU1350	CATGGCGTGATCCCTGACAT	GCCGAACGTGTTGAAGTAGG
CINAU1351	CTCGTCTTCCAGGCCTACC	GAGGTGGAAGTGGTCGAAGA
CINAU1354	GCGAGTACAACCTCATCGAC	CCTGTCCTTGCCCATCATCT

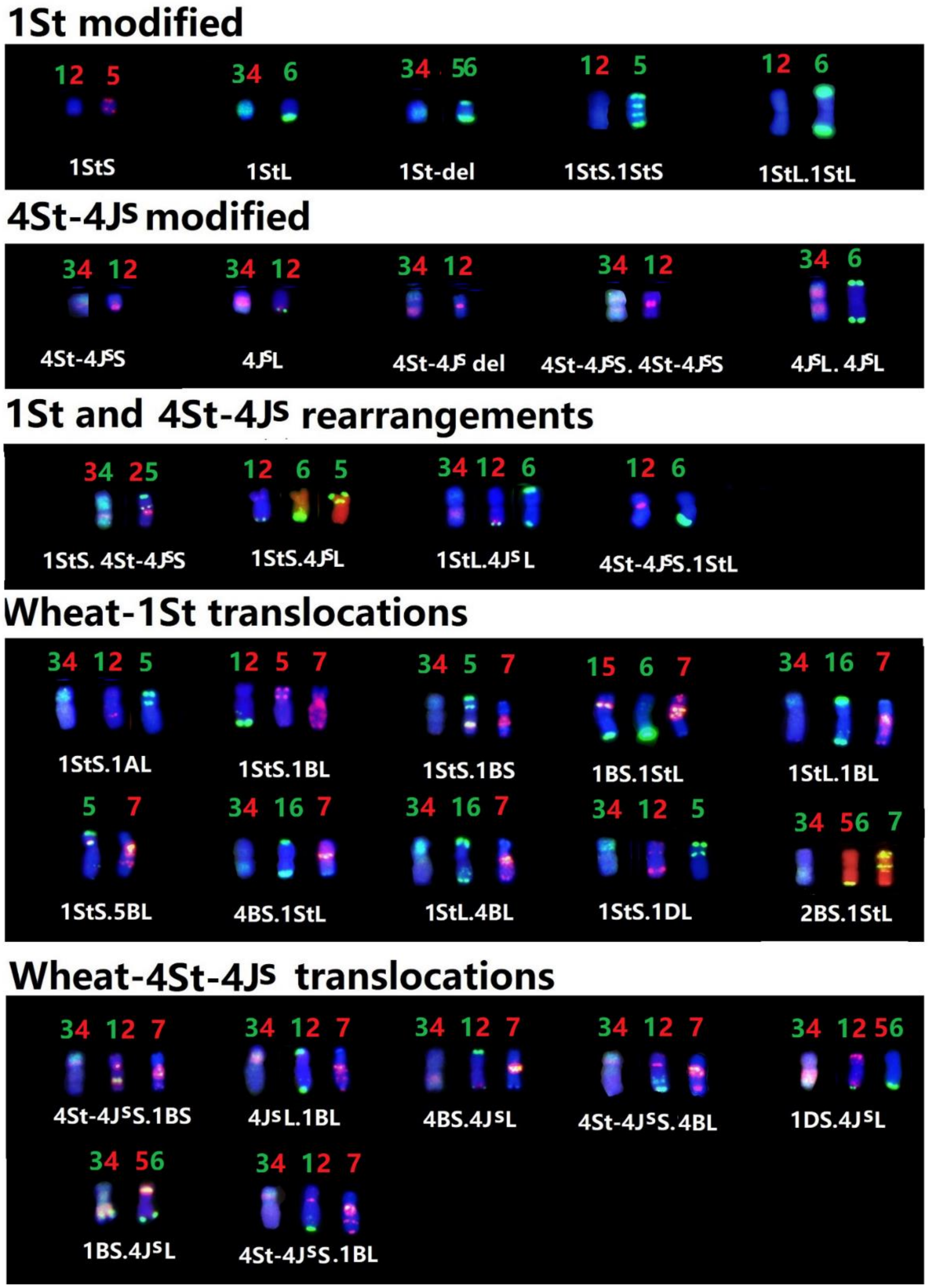

Figure S1. The representing chromosome translocations in the F_3 progenies of the hybrid between X479 and MY11.

The number 1-7 indicated the probes, (1) Oligo-pSc119.2, (2) Oligo-pTa535, (3) Oligo-pTa71, (4) Oligo-pSt122, (5) Oligo-B11, (6) Oligo-pDb12H, and (7) Oligo-(GAA)7, respectively.

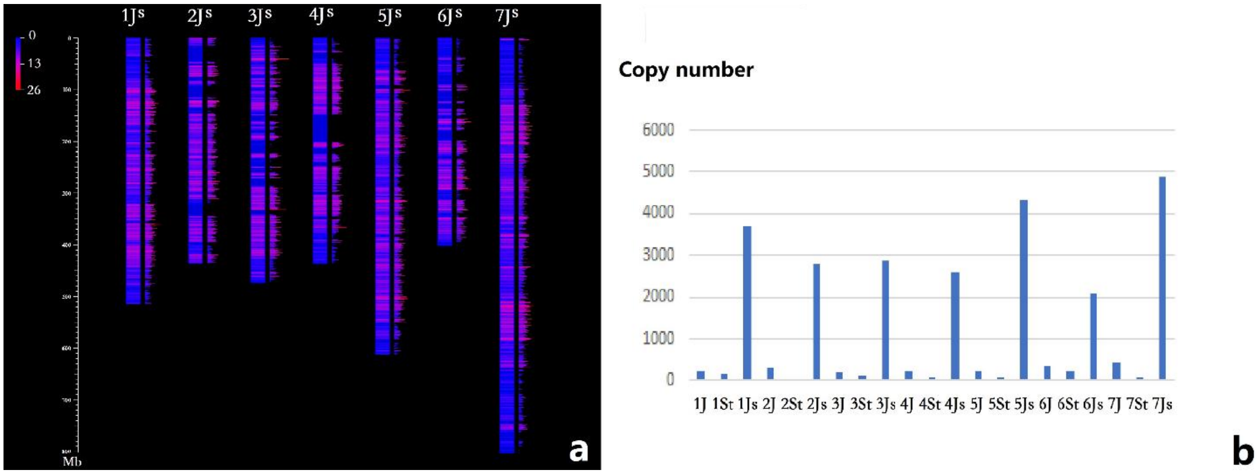

Figure S2. Distribution of Oligo-pDb12H predicted on genome of *Thinopyrum intermedium*

(a) The physical location of distribution of Oligo-pDb12H on J^S genome, (b) the copy number comparison of St, J, J^S chromosomes of Th. intermedium genome V2.1.

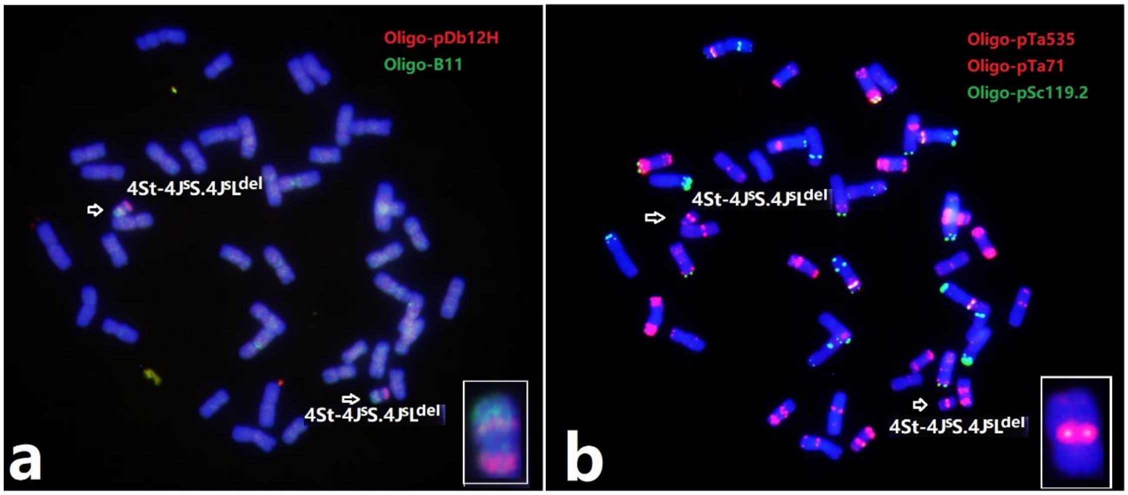

Figure S3. Sequential ND-FISH patterns of wheat-*Th. intermedium* deletion line M931. The probes for FISH were (a) Oligo-B11 (green) + Oligo-pDb12H (red), (b) Oligo-pSc119.2 (green) + Oligo-pTa535 (red) + Oligo-pTa71 (red). The cut-pasted chromosomes are 4St-J^{S}S.4J^{S}L^{del}.
